# Rapid-recovery protocol for minimally invasive mitral valve repair

**DOI:** 10.1016/j.xjon.2024.08.006

**Published:** 2024-08-23

**Authors:** Amy Brown, Ali Fatehi Hassanabad, Jolene Moen, Karen Wiens, Alexander J. Gregory, Ken Kuljit S. Parhar, Corey Adams, William D.T. Kent

**Affiliations:** a Department of Cardiac Sciences, Libin Cardiovascular Institute, University of Calgary, Calgary, Alberta, Canada; b Department of Anesthesiology, Libin Cardiovascular Institute, University of Calgary, Calgary, Alberta, Canada; c Department of Critical Care Medicine, Libin Cardiovascular Institute, University of Calgary, Calgary, Alberta, Canada

**Keywords:** minimally invasive valve, rapid recovery, early recovery after surgery, enhanced recovery, fast-track

## Abstract

**Background:**

Minimally invasive mitral valve repair (MIMVR), often performed within specialized care pathways, has been shown to reduce hospital length of stay and improve patient recovery. The relative value of rapid-recovery protocols as a component of care pathways, including enhanced recovery programs (ERPs), has not been well described. This study compared clinical outcomes following implementation of a new, comprehensive rapid-recovery protocol within a previously established, mature ERP for patients undergoing MIMVR.

**Methods:**

The rapid-recovery protocol was developed and implemented by a multidisciplinary team to further optimize patient recovery within an existing ERP. The protocol was applied to 75 consecutive patients undergoing MIMVR between September 2022 and December 2023. Outcomes were compared retrospectively to 75 ERP control patients who did not receive the rapid-recovery protocol but experienced the ERP. The primary outcome was a composite of discharge from the intensive care unit (ICU) by postoperative day (POD) 1, discharge to home by POD 4, and no all-cause hospital readmission by 30 days.

**Results:**

Baseline characteristics were similar in the 2 groups. Patients in the rapid-recovery group achieved the primary composite outcome significantly more often compared to the control group (60% vs 40%, respectively). There was no between-group difference in postoperative complications. Multivariable logistic regression showed that age ≤60 years was significantly associated with rapid-recovery protocol success. Clinical barriers to achieving individual components of the primary outcome were described.

**Conclusions:**

A rapid-recovery protocol for MIMVR was associated with early ICU and hospital discharge. These benefits were safely achieved without any increase in hospital readmission, morbidity, or mortality up to 30 days postoperatively.

**Figure:**
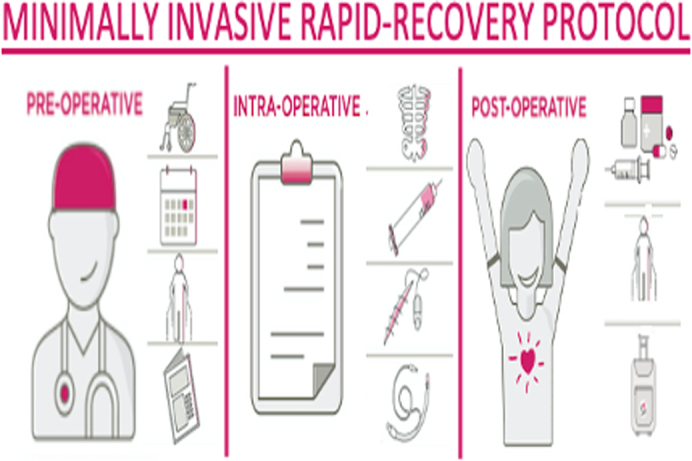
Rapid-recovery protocol for minimally invasive mitral valve repair.

**Central Message**

A rapid-recovery protocol for minimally invasive mitral valve repair promoted early intensive care and hospital discharge without hospital readmission or an increase in morbidity or mortality.

**Perspective**

The additive value of a comprehensive rapid-recovery protocol for minimally invasive mitral valve repair patients already following an enhanced recovery protocol (ERP) is unclear. A novel, minimally invasive–focused rapid-recovery protocol may improve clinical outcomes beyond to those achieved by an ERP.

Perioperative care in cardiac surgery has undergone considerable change to address the difficult balance between demand and available resources.^1^ To safely improve the efficiency of in-hospital patient recovery, enhanced recovery protocols (ERPs) have been developed.^2^ Along with promoting safe and efficacious recovery, a secondary goal is to decrease health resource waste.^1^^,^^3^

ERPs, formalized by the Enhanced Recovery After Surgery (ERAS) Society, are multidisciplinary approaches at the preoperative, intraoperative, and postoperative stages aimed at reducing the physiologic and psychological stress of surgery.^2^, ^3^, ^4^, ^5^ In noncardiac surgeries, these evidence-based recommendations have resulted in significant improvements in health resource utilization.^2^^,^^3^ The ERAS Society has published guidelines specific to cardiac surgery with promising results, but institutional uptake has varied.^2^, ^3^, ^4^ Recently, a joint expert consensus statement provided further strategies for optimal management of cardiac surgery patients.^2^

In addition to preoperative and postoperative care, the surgical approach also has been shown to influence clinical outcomes and health resource use. Minimally invasive mitral valve repair (MIMVR) has advantages over traditional open sternotomy, including decreased blood loss and reduced hospital length of stay (LOS).^6^^,^^7^

Within our institution, all cardiac surgery patients follow an ERP. Without the early functional limitations of sternotomy, MIMVR patients were hypothesized to have a greater potential for earlier gains and to potentially benefit from additional recommendations, facilitating more efficient recovery. As such, the goal of the present study was to compare outcomes before and after implementation of a comprehensive rapid-recovery protocol created specifically for elective MIMVR patients with degenerative disease. Our novel, comprehensive MIMVR-focused rapid-recovery protocol addresses each stage of the patient experience, with recommendations specific to this patient population.

## Methods

### Ethics Statement

This study was approved by the Conjoint Health Research Ethics Board at the University of Calgary underlying the Declaration of Helsinki (Research Ethics Board identification 22-0602; approved June 7, 2023). Patient consent was waived owing to the quality improvement nature of the study.

### Study Design and Patient Population

This study included 2 comparative groups: (1) patients in whom the rapid-recovery protocol was applied and (2) patients treated without the new rapid-recovery protocol. All patients underwent MIMVR for degenerative mitral valve disease and followed the preexisting ERP. The rapid-recovery protocol was established at our institution in September 2022. All patients who underwent elective MIMVR between September 2022 and December 2023 were included in the rapid-recovery group, totaling 75 consecutive patients. The control group comprised 75 consecutive patients who underwent MIMVR between May 2021 and August 2022, to achieve an equal number of patients in each group. Data were collected prospectively and compared to retrospectively collected data for the control group. Patients who required concomitant procedures, including left atrial appendage occlusion, atrial septal defect closure, or patent foramen ovale closure, were included in the study. Given that the patients were previously accepted for elective MIMVR, patients who required concomitant bypass surgery were excluded previously. Patients who had undergone previous cardiac surgery of any type were excluded. Patients who underwent concomitant procedures, such as maze or cryoablation, were excluded so as to keep the cohorts as homogenous as possible.

### Surgical Procedure

The patients in both cohorts had undergone MIMVR through a right fourth interspace mini-thoracotomy approach using single-lumen ventilation. Cannulation for cardiopulmonary bypass (CPB) was achieved peripherally through a 17 Fr or 19 Fr cannula in the femoral artery and a 25 Fr cannula in the femoral vein via surgical cutdown. No graft material was used for cannulation. A preoperative computed tomography scan was performed for all patients and the inclusion criteria for peripheral CPB was a common femoral artery >5.0 cm. A rib spreader was used for all patients after placement of a soft tissue retractor. Normothermia was maintained. The surgical technique for mitral valve repair was at the discretion of the operating surgical team and performed by 1 of 2 surgeons.

### Preexisting ERP

The rapid-recovery protocol was added to a preexisting and mature ERP. This included a preadmission clinic consultation with a cardiac anesthesiologist, a physiotherapist, and a social worker for optimization of medical conditions, patient education, and counseling on smoking/alcohol cessation. Patients were instructed to consume a small snack at 8 hours and clear fluids at 3 hours before surgery.

Multimodal analgesia was provided during all phases of care. The preoperative multimodal analgesia protocol according to the ERP and available to all patients in this study included oral acetaminophen 1000 mg and spinal anesthesia with administration of intrathecal morphine (150-300 μg) or acetaminophen and controlled-release (CR) oral hydromorphone 6 mg in patients age <75 years and weighing >60 kg or CR oral hydromorphone 3 mg in patients age >75 years or weighing <60 kg. Approximately 30% of the patients consented for acetaminophen and spinal anesthesia, and the other 70% received acetaminophen and hydromorphone perioperatively. Intraoperatively, ketamine, lidocaine, dexamethasone, magnesium, and dexmedetomidine were administered to selected patients. Postoperatively, nearly 100% of patients received some opioids, but this dropped to 20% by postoperative day (POD 3) and to <5% by discharge. Pain was scored on a visual analog scale of 0 to 10 both at rest and with activity. A score >3 was considered to indicate moderate or greater pain, for which patients were offered a low-dose, long-acting opioid (CR oral hydromorphone 6 mg for patients age <75 years and weighing >60 kg or CR oral hydromorphone 3 mg for patients age >75 or weighing <60 kg) in addition to regularly scheduled acetaminophen, supplemented with nonsteroidal anti-inflammatory drugs for patients with normal renal function.

All patients received pharmacologic prevention for postoperative nausea and vomiting, with high-risk patients receiving additional prophylaxis with aprepitant. Intraoperative lung protective mechanical ventilation strategies were applied. Intraoperative neuromonitoring, including cerebral oximetry and electroencephalography, was performed in all patients. Postoperatively, a formalized sedation weaning and extubation protocol was applied, with the goal of extubation in <6 to 8 hours. This ERP was implemented for all MIMVR patients beginning in July 2019.

### Rapid-Recovery Protocol

**Figure 1:**
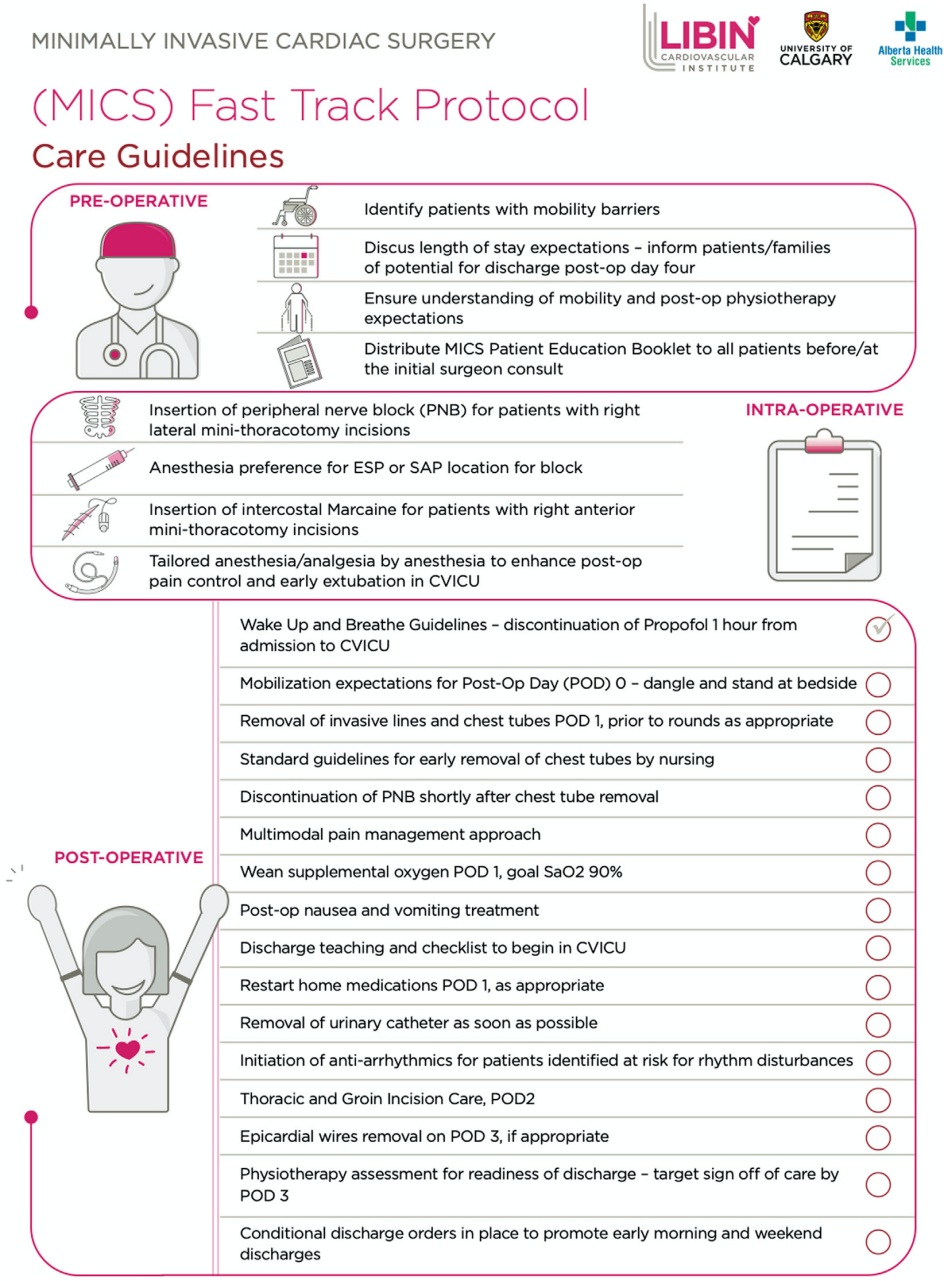
Abbreviated minimally invasive rapid-recovery protocol.

**Figure E1:**
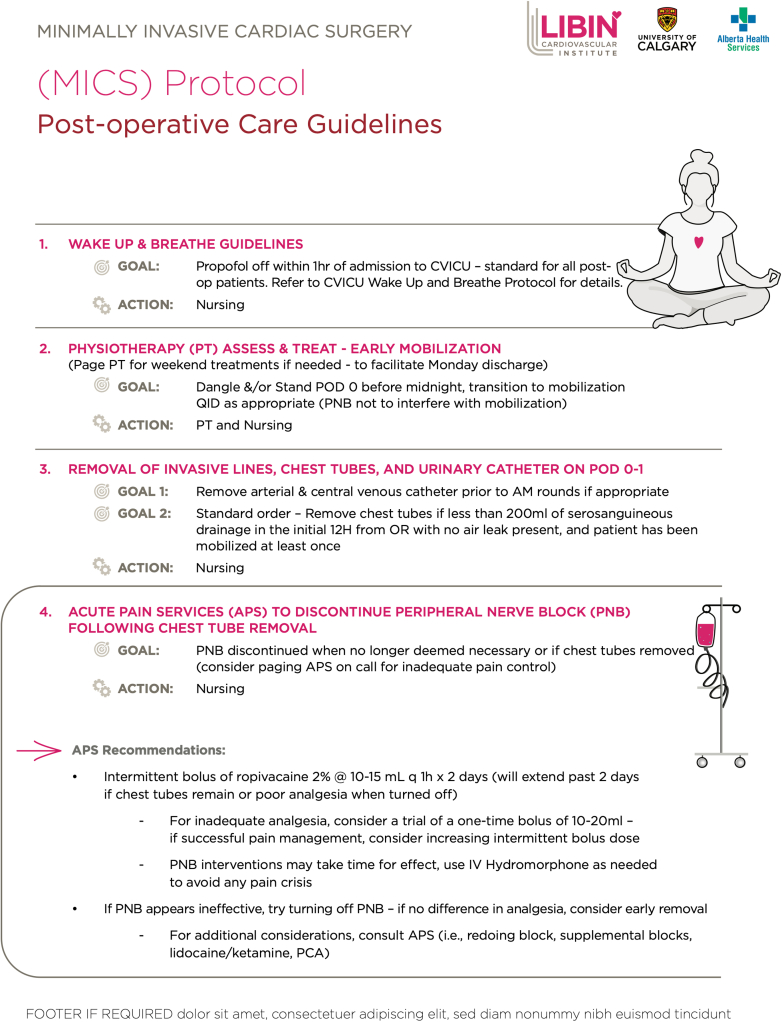

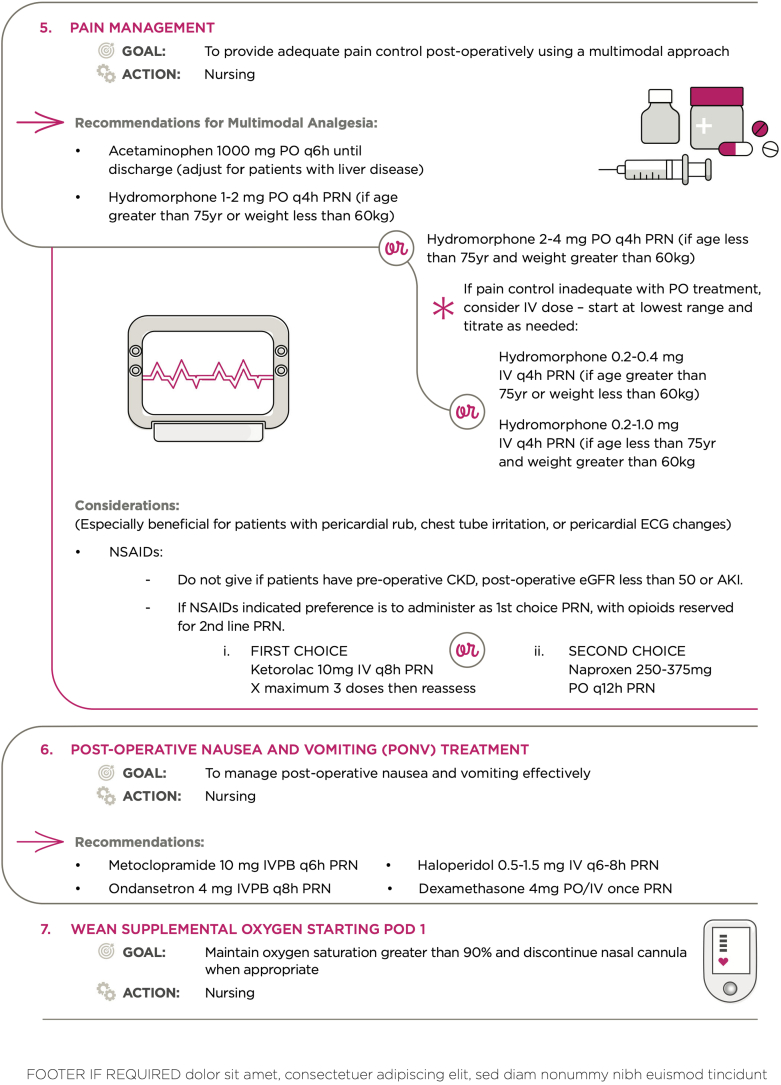

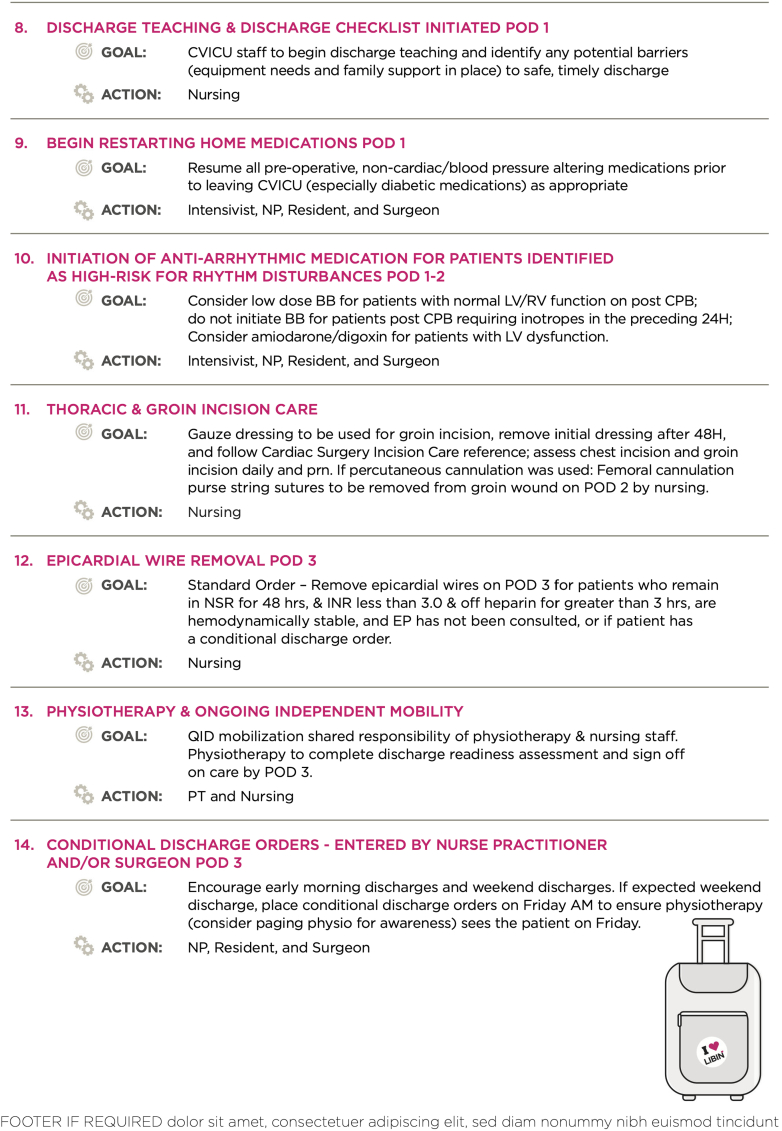
Comprehensive minimally invasive rapid-recovery protocol.

The main motivation for a formalized rapid-recovery protocol was to promote a cohesive team approach for postoperative care of MIMVR patients. The protocol was designed by a multidisciplinary team composed of physicians, nurses, physiotherapists, and other allied health professionals (Figure 1 and Figure E1). It was implemented for all patients who underwent MIMVR after September 2022. In addition to the existing ERP elements, rapid-recovery protocol patients had new care elements applied, including supplemental preadmission clinic discussion and an information booklet about patient and care team expectations after minimally invasive surgery. Perioperative multimodal analgesia was further augmented with standard use of erector spinae plane catheters for pain management. Postoperatively, an increased emphasis was placed on discontinuation of sedation, early extubation in <4 to 6 hours, and early removal of lines, tubes, and drains as benchmark process measures. Additional rapid-recovery protocol milestones included mobilization by the bedside nurse or physiotherapist before midnight on the day of intensive care unit (ICU) admission following surgery. Chest tubes, central and arterial lines, and urinary catheters were to be removed by POD 1. Transfer to the cardiac surgery ward was recommended by POD 1. Discharge teaching was initiated by POD 1 to ensure that the patient had clear expectations for recovery and care progress. Postoperative pain management was primarily non–opioid-based, with erector spinae plane blocks managed by the Acute Pain Services team.

### Primary Outcomes

The primary outcome was a composite of (1) discharge from the ICU by POD 1, (2) discharge to home by POD 4, and (3) no 30-day all-cause readmission. These parameters were chosen based on previously reported outcome measures and were designed to provide a high-level overview of clinical outcomes, as well as a balancing measure of readmission.^6^^,^^8^

### Secondary Outcomes

Secondary outcomes were intubation time, ICU LOS, hospital LOS, and postoperative complications at 30 days, including acute kidney injury, atrial fibrillation, bleeding necessitating transfusion or return to the operating room, death, delirium, pleural effusion necessitating intervention, and stroke. The time of mechanical ventilation started at ICU admission to prevent confounding by procedure time.

### Statistical Analyses

Continuous variables are reported as mean ± SD, with significance determined by the Kruskal-Wallis test. Categorical variables are reported as frequency with corresponding percentage, with significance determined using the Fisher exact test. Logistic regression was performed to identify factors associated with rapid-recovery success. Effect sizes for regression analysis are expressed as odds ratio (OR) with corresponding 95% confidence interval (CI). A 2-sided α level of 0.05 defined statistical significance. Statistical analysis was performed using Stata version 17.0 (StataCorp).

## Results

### Baseline Characteristics

The study comprised a total of 150 patients, including 75 in the rapid-recovery group and 75 in the control group. Baseline characteristics, based on preoperative diagnoses and self-reported gender, were similar in the 2 groups, except for significantly higher rates of dyslipidemia and diabetes mellitus in the control group. A detailed overview of clinical baseline characteristics is provided in Table 1.

**Table 1:** Baseline patient characteristics

Characteristic	Preprotocol (N = 75)	Postprotocol (N = 75)	*P* value
Age, y, mean ± SD	60.4 ± 12.1	59.9 ± 12.8	.987
Female sex, n (%)	30 (40.0)	26 (34.7)	.613
BMI, kg/m^2^, mean ± SD	26.5 ± 4.8	26.4 ± 4.5	.951
NYHA class III-IV, n (%)	13 (17.3)	19 (25.3)	.319
Hypertension, n (%)	32 (42.7)	27 (36.0)	.504
Dyslipidemia, n (%)	47 (62.7)	31 (41.3)	**.014**
Coronary artery disease, n (%)	15 (20.0)	10 (13.3)	.381
Diabetes, n (%)	10 (13.3)	1 (1.3)	**.009**
Smoking, n (%)	8 (10.7)	5 (6.7)	.563
COPD, n (%)	2 (2.7)	2 (2.7)	1.000
Chronic kidney disease, n (%)	3 (4.0)	5 (6.7)	.719
Previous stroke or TIA, n (%)	5 (6.7)	0 (0.0)	.058
Atrial fibrillation, n (%)	17 (22.7)	12 (16.0)	.409
LVEF ≥60%, n (%)	58 (77.3)	61 (81.3)	.687
Tricuspid regurgitation, n (%)			
None/trivial	55 (73.3)	57 (76.0)	.851
Mild	17 (22.7)	15 (20.0)	.842
Moderate	3 (4.0)	4 (5.3)	1.000
Severe	0 (0)	0 (0.0)	1.000
Aortic regurgitation, n (%)			
None/trivial	68 (90.7)	63 (84.0)	.326
Mild	6 (8.0)	11 (14.7)	.303
Moderate	1 (1.3)	1 (1.3)	1.000
Severe	0 (0.0)	0 (0.0)	1.000

Bold type indicates statistical significance. *BMI*, Body mass index; *NYHA*, New York Heart Association; *COPD*, chronic obstructive pulmonary disease; *TIA*, transient ischemic attack; *LVEF*, left ventricular ejection fraction.

### Primary Outcome

**Figure 2:**
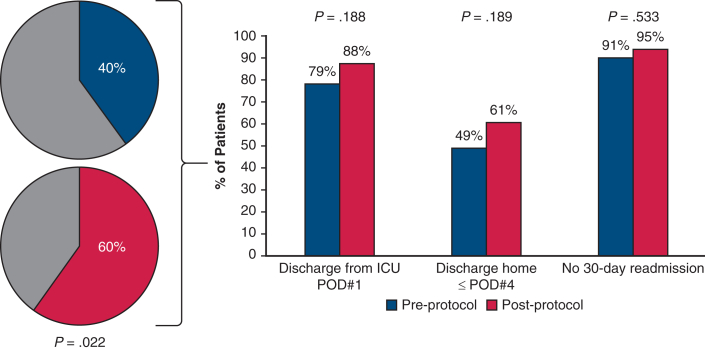
Primary composite outcome and individual components of primary outcome comparing preprotocol and postprotocol cohorts. *ICU*, Intensive care unit; *POD*, postoperative day.

Overall, 45 patients (60.0%) were found to meet all components of the composite primary outcome in the rapid-recovery group, compared to 30 patients (40.0%) in the control group (*P* = .022). All individual outcomes within the composite were increased in the rapid-recovery group, but the differences did not reach significance (Figure 2). Follow-up was complete for 100% of patients.

### Secondary Outcomes

There were no significant differences in operative data, postoperative data, or postoperative outcomes at 30 days between the control and rapid-recovery groups (Table 2). The median duration of intubation was 5.4 hours (interquartile range [IQR], 4.4-9.6 hours) in the control group compared to 6.6 hours (IQR, 4.4-11.2 hours) in the rapid-recovery group (*P* = .296). Sex-specific analyses showed no difference in intubation time, ICU LOS, or hospital LOS; however, there was a trend toward longer mean hospital LOS in female patients compared to male patients (6.2 ± 4.8 days vs 4.8 ± 2.4 days; *P* = .102). Overall, 17% of patients postprotocol were discharged to home by POD 3, compared to 7% in the preprotocol cohort.

**Table 2:** Secondary outcomes in the study cohorts

Clinical outcome	Preprotocol (N = 75)	Postprotocol (N = 75)	*P* value
Operative data			
Ring annuloplasty, n (%)	73 (97.3)	72 (96.0)	1.000
Neochord placement, n (%)	37 (49.3)	50 (66.7)	.047
Leaflet resection, n (%)	0 (0.0)	4 (5.3)	.120
CPB time, min, median (IQR)	113 (96-126)	110 (95-131)	.803
Aortic cross-clamp time, min, median (IQR)	84 (71-93)	84 (70-102)	.769
Residual MR ≥ mild, n (%)	9 (12.0)	6 (8.0)	.422
Postoperative data			
Intubation time, h, median (IQR)	5.4 (4.4-9.6)	6.6 (4.4-11.2)	.265
ICU LOS, d, median (IQR)	1 (1-1)	1 (1-1)	.295
Postoperative hospital LOS, d, median (IQR)	5 (4-6)	4 (4-5)	.230
ICU readmission, n	0	0	-
Reintubation, n	0	0	-
Pacemaker, n (%)	1 (1.3)	3 (4.0)	.620
Complications, n (%)			
Acute kidney injury	0	0	-
Atrial fibrillation	29 (38.7)	22 (29.3)	.301
Bleeding	0	0	-
Death	0	0	-
Delirium	0	3 (4.0)	.245
Pleural effusion	5 (6.7)	4 (5.3)	1.000
Hospital readmission	7 (9.3)	4 (5.3)	.533
Stroke	0	0	-

*CPB*, Cardiopulmonary bypass; *IQR*, interquartile range; *MR*, mitral regurgitation; *ICU*, intensive care unit; *LOS*, length of stay.

### Factors Associated With Rapid-Recovery Protocol Success

Univariable logistic regression of preoperative and operative variables showed that age ≤60 years (OR, 4.53; 95% CI, 1.65-12.42; *P* = .003) and trivial or less preoperative tricuspid regurgitation trivial (OR, 3.14; 95% CI, 1.05-9.40; *P* = .04) were significantly associated with rapid-recovery success. In the multivariable logistic regression of these significant variables and rapid-recovery success, only age ≤60 years maintained statistical significance (OR, 3.64; 95% CI, 1.27-10.42; *P* = .016) (Table 3). More patients age >60 years were female or had mild aortic insufficiency or mild tricuspid regurgitation.

**Table 3:** Multivariable regression analysis to identify variables associated with rapid-recovery success

Variable	Univariable		Multivariable	
	OR (95% CI)	*P* value	OR (95% CI)	*P* value
Age ≤60 y	**4.53 (1.65-12.42)**	**.003**	**3.64 (1.27-10.42)**	**.016**
Female sex	0.42 (0.16-1.10)	.078	-	-
BMI (kg/m^2^)	1.00 (0.84-1.12)	.863	-	-
NYHA III-IV	0.38 (0.13-1.05)	.063	-	-
Hypertension	1.21 (0.46-3.20)	.695	-	-
Dyslipidemia	0.57 (0.22-1.48)	.245	-	-
Coronary artery disease	0.63 (0.16-2.38)	.491	-	-
Diabetes	1	-	-	-
Smoking	1.00 (0.16-6.37)	1.000	-	-
COPD	1	-	-	-
Chronic kidney disease	0.42 (0.07-2.67)	.357	-	-
Previous stroke or TIA	1	-	-	-
Atrial fibrillation	0.62 (0.18-2.13)	.443		-
LVEF ≥60%	1.16 (0.36-3.75)	.809	-	-
Tricuspid regurgitation ≤ trivial	**3.14 (1.05-9.40)**	**.04**	2.07 (0.64-6.76)	.226
Aortic regurgitation ≤ trivial	2.43 (0.69-8.56)	.165	1.62 (0.41-6.38)	.493
CPB time	0.99 (0.97-1.01)	.339	-	-
Aortic cross-clamp time	1.00 (0.97-1.02)	.697	-	-
Additional procedure∗	0.32 (0.09-1.21)	.094	-	-
Residual MR ≤ trivial	0.73 (0.13-4.27)	.729	-	-

Bold type indicates statistical significance. *OR*, Odds ratio; *CI*, confidence interval; *BMI*, body mass index; *NYHA*, New York Heart Association; *COPD*, chronic obstructive pulmonary disease; *TIA*, transient ischemic attack; *LVEF*, left ventricular ejection fraction; *CPB*, cardiopulmonary bypass.

∗ Patent foramen ovale closure, or atrial septal defect closure, or removal of left atrial appendage.

### Barriers to Rapid-Recovery Protocol Success

The most common reason that patients on the rapid-recovery group were not discharged from the ICU by POD 1 was a low-output state, defined by an increased serum lactate level necessitating inotropic support, albeit affecting only 5.3% of the postprotocol group. Thirty-nine percent of the postprotocol group could not be discharged to home by POD 4, with postoperative atrial fibrillation as the most common reason reported. Only 5% of patients were readmitted within 30 days of their surgery, owing mostly to pleural effusions necessitating drainage. Table 4 provides further details on barriers to rapid-recovery success.

**Table 4:** Patient-associated barriers to achieving individual components of the primary outcome

Barriers to achieving primary outcome	Affected patients, n (%)
ICU POD 1 discharge	
Delirium	1 (1.3)
Hemodynamic instability	2 (2.7)
Low-output state	4 (5.3)
Rhythm	1 (1.3)
Seizure	1 (1.3)
Hospital discharge by POD 4	
Delirium	2 (2.7)
Mobility	4 (5.3)
Myocardial infarction	1 (1.3)
Rhythm control	
Atrial fibrillation	8 (10.7)
Pacemaker insertion	3 (4.0)
Respiratory	
Oxygen requirement	4 (5.3)
Pneumonia	1 (1.3)
Pneumothorax	1 (1.3)
Pain	4 (5.3)
Reason for 30-d readmission	
Pericarditis	1 (1.3)
Pleural effusion	2 (2.7)
Gastrointestinal bleed	1 (1.3)

*ICU*, Intensive care unit; *POD*, postoperative day.

## Discussion

This comprehensive rapid-recovery protocol implemented specifically for patients after MIMVR was associated with significantly higher rates of the primary composite outcome of discharge from the ICU by POD 1, discharge to home by POD 4, and no readmission by 30 days. These clinical outcomes were achieved safely, with no difference in postoperative complications between the 2 study groups. This novel comprehensive minimally invasive–focused rapid-recovery protocol was applied to a surgical population for which ERAS protocols are standard of care. These findings suggest that a combination of the minimally invasive approach, ERPs, and the rapid-recovery protocol can improve patient recovery.

ERAS ERPs used in cardiac surgery have been associated with reduced ICU and hospital LOS; however, the mean reported hospital LOS is still 6 to 8 days.^8^, ^9^, ^10^ Earlier discharge strategies in the context of minimally invasive valve surgery have shown that discharge within 3 days of surgery is safe and cost-effective.^8^ This suggests that there is an opportunity to improve the patient experience by facilitating a more efficient recovery. Our rapid-recovery protocol for MIMVR may serve as a tool to realize this potential. A similar study on the feasibility of early hospital discharge after robotic cardiac surgery showed that discharge on POD 1 or 2 was associated with lower morbidity and similar readmission and mortality rates compared to patients discharged on POD 3 or later.^11^ Their results are attributed to efforts to foster a culture amenable to early discharge, as well as to framing readiness for discharge in a patient-centered approach while achieving standard postoperative milestones.^11^ Therefore, instituting such a culture change with a rapid-recovery protocol, even in the context of a mature ERP, likely can promote better recovery for patients while improving cost-effectiveness in the healthcare system.^8^^,^^11^

Surgery for mitral valve disease has become increasingly less invasive over the past decade, with reported benefits including less blood transfusions and less time in the hospital.^7^ Notably, our protocol was associated with benefits beyond those attributed solely to the minimally invasive approach. However, an important aspect to consider is selection bias, as the included patients were generally younger with fewer comorbidities compared to patients requiring other types of cardiac surgical intervention. It may be that this surgical population is more responsive to enhanced initiatives or that their preoperative characteristics predispose them to a comparatively faster recovery.

An additional consideration is the technical learning curve for minimally invasive surgery. There is a trend toward improved complication rates after the initial 100 procedures, with reduced aortic cross-clamp and CPB times and lower rates of perioperative death, stroke, myocardial infarction, bleeding, vascular complications, acute kidney injury, conduction disturbances, and valve-related complications.^12^ MIMVR has been offered at our institution since 2012, with both surgeons involved in this study (C.A. and W.D.T.K.) having performed approximately 100 cases each per year since 2017. As such, they are considered to be past the documented technical learning curve.^12^

It is unlikely that one specific recommendation within the protocol is singularly responsible for any benefits; instead, benefits accrue incrementally in many steps throughout the patient’s course of care.^9^^,^^11^ To capture any cumulative benefit of the protocol, we used a composite outcome for the primary outcome instead of single measures. The aim was to encompass important components of the postoperative experience, using ICU and hospital discharge, as well as a safety indicator of no 30-day readmission.

Because cardiac surgery patients usually convalesce initially in the ICU, time to extubation can be considered a marker of progression and serves as an indicative measurement of the entire perioperative period.^13^^,^^14^ Time to extubation might not be the rate-limiting step in patient recovery, however. Our results show that intubation time did not change significantly after implementation of the rapid-recovery protocol, and yet patients in the protocol group had a significantly higher rate of the primary composite outcome. Moreover, patients in the protocol group had a trend toward quicker discharge from the hospital, although the difference was not significantly different. Therefore, whether a goal of shorter intubation time could translate into a clinical advantage is unclear. Some reports on early extubation protocols have shown an increased ICU LOS in patients extubated <6 hours after cardiac surgery, as well as increased rates of reintubation and other postoperative complications.^15^^,^^16^ Other reports have demonstrated that patients who were extubated either in the operating room or within 6 hours of cardiac surgery were more likely to have a reduced hospital LOS.^17^ A Cochrane meta-analysis of fast-track protocols in cardiac surgery demonstrated that time-directed extubation protocols reduced intubation time and ICU LOS but did not impact hospital LOS or postoperative complications.^6^ Therefore, extubation within 6 hours of surgery appears to be safe, but the level of appreciable benefit is unclear.^5^ As such, we chose not to focus our rapid-recovery protocol on time-directed extubation.^18^ Future iterations of our protocol may include operating room extubation for selected patients undergoing MIMVR, based on accumulating evidence demonstrating the safety of operating room extubation post surgery.

Overall, 40% of the post protocol group did not achieve all components of the primary outcome. Multivariable logistic regression of preoperative and operative characteristics found that only age ≤60 years was significantly associated with the primary outcome. This is in contrast to a single retrospective cohort study of 491 patients after MIMVR that identified New York Heart Association class III-IV, chronic kidney disease, coronary artery disease, procedure time, and revision for bleeding as associated with fast-track course failure.^19^ It may be that our small sample size as well as low rates of adverse events did not allow for evaluation of these relationships. In our study, age may be a proxy for overall frailty that is not captured by other variables.

Regarding postoperative variables impacting the rate of the primary outcome, 12% of post protocol patients were not discharged from the ICU by POD 1, mostly (5.3%) due to a temporary low-output syndrome necessitating ionotropic support, with no patients requiring mechanical support. The reported incidence of low-output syndrome after isolated mitral valve surgery ranges from 7% to 36%.^20^, ^21^, ^22^ Our lower rate likely was due to the selected patient population with few predictive risk factors associated with the development of low-output syndrome.^22^ Thirty-three percent of post protocol patients were not discharged to home by POD 4, with postoperative atrial fibrillation the most common reason. A recent ERAS/Society of Thoracic Surgeons consensus statement recommends a multifaceted prevention strategy.^2^ Our rapid-recovery protocol recommends use of a beta-blocker in patients with normal biventricular function and amiodarone or digoxin in patients with left ventricular dysfunction by POD 1 or 2. Further iterations of our protocol may include prophylactic use of beta-blockers and amiodarone or other preventive strategies to attempt to reduce this barrier to successful rapid-recovery protocol trajectory.^23^ Although no patients were readmitted due to postoperative atrial fibrillation, it is possible that new-onset postoperative atrial fibrillation might have occurred after early discharge. Before discharge, all patients are counseled on reasons to seek medical care, including rapid heart rate and palpitations. Furthermore, follow-up with their primary care physician within 2 weeks of surgery and their cardiologist within 6 weeks from surgery is recommended for all patients. Few (5.3%) post protocol patients required hospital readmission for any reason. Thus, maintaining a highly selected patient population with consideration of postoperative management of atrial fibrillation may promote success with future iterations of our rapid-recovery protocol or for other centers keen on adopting this protocol.

Implementing change within an institution is not easy, particularly in cardiac surgery, a discipline that relies on consistency. Any innovation must be implemented with the support of numerous stakeholders, including healthcare providers, administration, and patients.^24^^,^^25^ From its conception, our protocol relied on multidisciplinary input from physicians, nurses, respiratory therapists, physiotherapists, and administrators. This approach was key to the successful design and adoption within the institution, as stakeholders had shared responsibility. The protocol was introduced to physicians, nurses, and allied health professionals at departmental rounds. During the first several weeks after the protocol was implemented, a clinical nurse educator at the bedside followed each patient through the clinical pathway.

Moreover, institutional change does not end once the change has been implemented. Continued assessment of the change to ensure that the desired result is attained without compromising patient safety is paramount.^24^^,^^25^ For instance, our team held quarterly meetings to identify patient and provider barriers to adoption of the rapid-recovery protocol. This led to improved optimization of the protocol through revised recommendations and strategies for improving staff awareness and engagement. Further team awareness was achieved by the development of a data dashboard, allowing team members to readily analyze outcomes, processes, and balancing metrics while highlighting the progress of protocol implementation.

Our conclusions need to be interpreted in light of several limitations of the study. First, the design was observational and thus cannot determine causation. Regarding the logistic regression, a post hoc power analysis found the power to be 69% based on the incidence of the primary outcome (40% and 60%) and 75 participants in each group, with an α level of 0.05. The interpretation of this value is unclear, as a post hoc power analysis has been found to be an invalid method of addressing validity and reproducibility.^26^ This is because the post hoc power is a deterministic function of the *P* value generated from the test, meaning that if the *P* value exceeds the α level of .05, then the post hoc power will always be <50%. Therefore, a post hoc power analysis does not indicate whether a larger sample size is warranted; however, a prestudy power analysis could make this determination. Additionally, our small patient population was highly specific and focused on one type of surgical procedure. Although this avoids confounding by disease and surgical procedure, it limits the generalizability of our results until outcomes from the rapid recovery protocol are reported from a larger, expanded patient population. Furthermore, the short follow-up precluded the analysis of any long-term effects. In addition, owing to the study’s retrospective nature, quality of life indicators could not be obtained. It would be worthwhile to include patient-reported outcome measures for quality of life in future studies of recovery protocols.

## Conclusions

This study shows that a comprehensive, minimally invasive–focused rapid-recovery protocol can facilitate earlier ICU and hospital discharge without readmission, providing improved patient recovery beyond that achieved solely with ERAS recommendations. Based on our early experience, we hypothesized that patients would show a benefit, with safely reduced time in the ICU and hospital. We suspect that with further study and expanded cohort size, we may see significant benefits for individual components of the composite outcome.

## Conflict of Interest Statement

The authors reported no conflicts of interest.

The *Journal* policy requires editors and reviewers to disclose conflicts of interest and to decline handling or reviewing manuscripts for which they may have a conflict of interest. The editors and reviewers of this article have no conflicts of interest.
